# Iron-responsive element-binding protein 2 plays an essential role in regulating prostate cancer cell growth

**DOI:** 10.18632/oncotarget.19288

**Published:** 2017-07-17

**Authors:** Zhiyong Deng, David H. Manz, Suzy V. Torti, Frank M. Torti

**Affiliations:** ^1^ Department of Molecular Biology and Biophysics, UCONN Health, Farmington, Connecticut 06032, USA; ^2^ School of Dental Medicine, UCONN Health, Farmington, Connecticut 06032, USA; ^3^ Department of Medicine, UCONN Health, Farmington, Connecticut 06032, USA

**Keywords:** prostate cancer, iron, iron-responsive element-binding protein, cell cycle, apoptosis

## Abstract

Iron-responsive element-binding proteins (IRPs) are master regulators of cellular iron homeostasis. Our previous work demonstrated that iron homeostasis is altered in prostate cancer and contributes to prostate cancer progression. Here we report that prostate cancer cells overexpress IRP2 and that overexpression of IRP2 drives the altered iron phenotype of prostate cancer cells. IRP2 knockdown in prostate cancer cell lines reduces intracellular iron and causes cell cycle inhibition and apoptosis. Cell cycle analysis demonstrates that IRP2-depleted prostate cancer cells accumulate in G0/G1 due to induction of p15, p21, and p27. Activation of these pathways is sufficient to significantly reduce the growth of PC3 prostate tumors *in vivo*. In contrast, IRP1 knockdown does not affect iron homeostasis and only modestly affects cell growth, likely through an iron-independent mechanism. These results demonstrate that upregulation of IRP2 in prostate cancer cells co-opts normal iron regulatory mechanisms to facilitate iron retention and drive enhanced tumor growth.

## INTRODUCTION

Iron is essential for normal cellular metabolism, growth, and replication. Iron and iron-containing functional groups, such as heme and iron-sulfur clusters, are critical to the function of proteins involved in numerous cellular processes including mitochondrial respiration, DNA synthesis, and cell cycle regulation [1]. Excess iron, however, is physiologically detrimental, contributing to generation of reactive oxygen species and oxidative stress [2]. As a result, cellular iron homeostasis is tightly controlled [3, 4].

Iron-responsive element-binding proteins (IRPs) are master regulators of cellular iron homeostasis. The two IRPs, IRP1 and IRP2, share 61% sequence identity [5]. Both IRPs post-transcriptionally regulate iron levels by binding to iron responsive elements (IREs), stem loop structures in the 5’ or 3’ untranslated regions (UTR) of selected mRNAs [6, 7]. IRP/IRE binding in the 5’ UTR suppresses mRNA translation while IRP/IRE binding in the 3’ UTR stabilizes mRNA transcripts and results in increased translation. 5’ IREs are found in mRNAs encoding proteins involved in iron storage and export, such as ferritin and ferroportin (FPN), while 3’ IREs are found in mRNAs encoding proteins involved in iron import, such as transferrin receptor (TfR1) and divalent metal transporter 1 (DMT1). Consequently, IRP/IRE binding is normally stimulated in low iron conditions and orchestrates an increase in cellular iron [1].

Prostate cancer is the second leading cause of cancer deaths among men in the United States [8]. Prostate tumors exhibit increased levels of intracellular iron, as do other cancers such as breast [9] and ovarian cancer [10]. Iron import proteins, including TfR1 [11], are overexpressed in prostate cancer cells, while iron export proteins, including FPN, are downregulated [12]. Increased iron retention driven by increased TfR1 and reduced FPN is consistent with increased IRP activity. We therefore hypothesized that IRP overexpression, which occurs in breast cancer [13], drives the altered iron phenotype observed in prostate cancer.

In this manuscript we demonstrate that overexpression of IRP2, but not IRP1, is a key mechanism by which prostate cancer cells accumulate intracellular iron to drive tumor growth. *In vitro*, IRP2 knockdown in prostate cancer cell lines reduces intracellular iron, inhibiting cell cycle progression by inducing cell cycle inhibitors, as well as triggering apoptosis. IRP2 knockdown also significantly reduces tumor growth *in vivo*.

## RESULTS

### Transferrin receptor 1 is increased and ferritin H is decreased in prostate cancer cells compared to normal prostate epithelia

**Figure 1:**
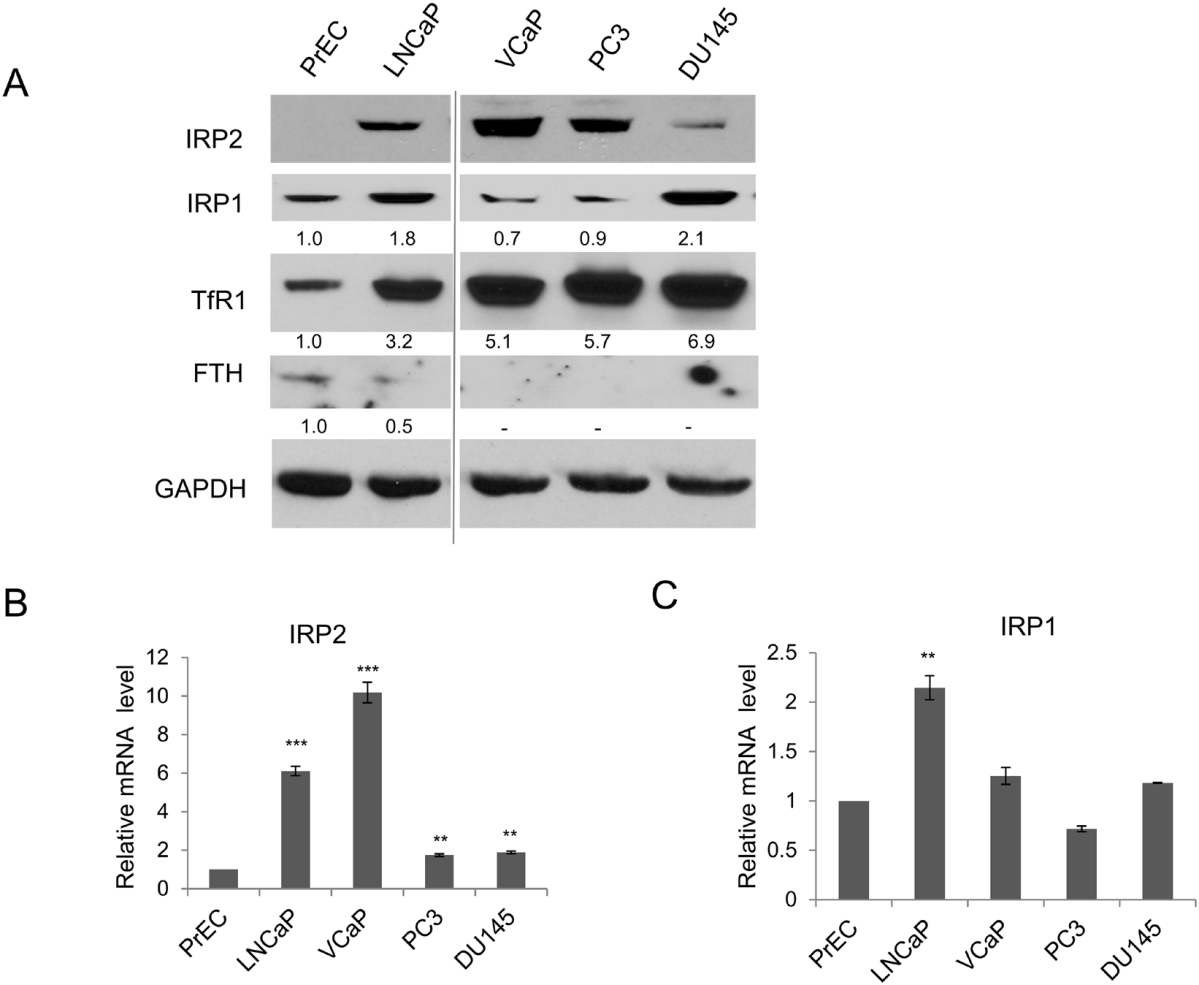
IRP2 is overexpressed in prostate cancer cells **(A)** Western blot of transferrin receptor 1 (TfR1), ferritin H (FTH) (monoclonal antibody detection), iron regulatory protein 1 (IRP1), iron regulatory protein 2 (IRP2), and GAPDH (loading control) in normal prostate epithelia (PrEC) and prostate cancer cells (LNCaP, VCaP, PC3, DU145). **(B, C)** Relative mRNA levels of (B) IRP2 and (C) IRP1 detected by real-time qPCR. Data are representative of 3 independent experiments (* p < .05, ** p < .01, *** p < .001).

Previous work has shown that prostate cancer cells retain higher levels of metabolically active iron, known as the labile iron pool (LIP), than benign prostate epithelia [12]. Two proteins important for modulation of intracellular iron levels are the iron import protein TfR1 and the iron storage protein ferritin. To determine if these proteins were altered in prostate cancer cells, we compared protein levels of TfR1 and ferritin H (FTH), a subunit of ferritin, in normal prostate epithelial cells (PrEC) and several prostate cancer cell lines (LNCaP, VCaP, PC3, and DU145). As illustrated in Figure 1A, TfR1 protein was markedly increased in all prostate cancer cell lines as compared to normal prostate epithelial cells. In contrast, FTH protein levels were decreased to barely detectable levels in prostate cancer cells. Elevated TfR1 and downregulated ferritin are associated with a high iron uptake and low iron storage capability.

### IRP2 is upregulated in prostate cancer cells

The increase in TfR1 and decrease in ferritin observed in prostate cancer cells appear to contradict what is known about iron regulatory mechanisms. In particular, in the classic model of iron regulation, high levels of intracellular iron are expected to inactivate iron regulatory proteins (IRP1 and IRP2), which in turn would decrease TfR1 and increase ferritin through post-transcriptional mechanisms [14]. However in prostate cancer cells, we observed a decrease in ferritin and increase in TfR1 (Figure 1), despite the increased labile iron pool in these cells [12]. To explain this paradox, we hypothesized that prostate cancer cells might constitutively over-express either IRP1 or IRP2, and that overexpression of these proteins might over-ride the classic iron-mediated iron regulatory pathway to permit high levels of TfR1 and low levels of ferritin in these iron replete cells.

To test this hypothesis, we measured levels of IRP1 and IRP2 in these cells. As shown in Figure 1A, IRP2 was consistently overexpressed in prostate cancer cell lines, with 4/4 cell lines exhibiting elevated levels of IRP2 when compared to normal prostate epithelial cells. In contrast, IRP1 protein levels were elevated in only two of four prostate cancer cell lines. Mirroring these changes at the protein level, mRNA levels of IRP2 were consistently upregulated in prostate cancer cells (Figure 1B). Levels of IRP1 were generally not increased, with only LNCaP cells exhibiting a modest increase in IRP1 (Figure 1C). Taken together, these results suggested IRP2 rather than IRP1 as a critical contributor to the altered iron phenotype of prostate cancer cells.

### IRP2 is critical for altered iron homeostasis in prostate cancer cells

**Figure 2:**
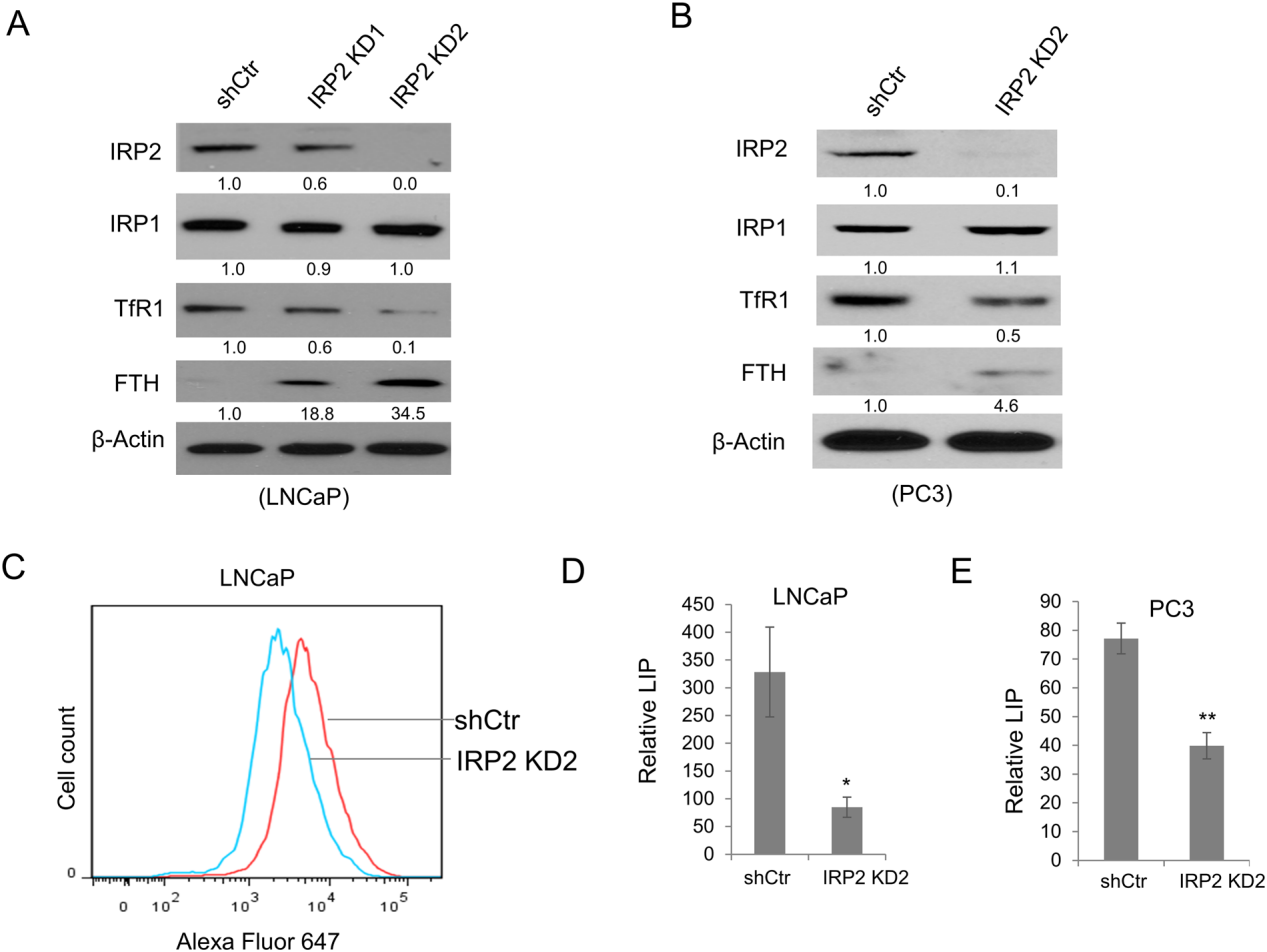
IRP2 knockdown alters prostate cancer iron metabolism and reduces intracellular iron level **(A, B)** Western blot of iron regulatory protein 2 (IRP2), iron regulatory protein 1 (IRP1), transferrin receptor 1 (TfR1), ferritin H (FTH) (monoclonal antibody detection), and β-actin (loading control) in (A) LNCaP and (B) PC3 cells infected with lentiviral IRP2-shRNA(s) (IRP2 KD) and scrambled control shRNA (shCtr). **(C)** Alexa Fluor647-conjugated transferrin uptake assessed by flow cytometry in shCtr and IRP2 KD LNCaP cells. **(D, E)** Labile iron pool (LIP) levels in (D) LNCaP and (E) PC3 shCtr and IRP2 KD cells. Data are representative of 3 independent experiments (* p < .05, ** p < .01).

To determine if IRP2 overexpression drives the iron phenotype of prostate cancer cells, we depleted IRP2 in LNCaP cells using two independent lentiviral shRNAs (Figure 2A). Following IRP2 knockdown, LNCaP cells expressed dramatically reduced TfR1 and increased FTH (Figure 2A). Importantly, we observed no effect of IRP2 shRNAs on IRP1 (Figure 2A). In PC3 cells, IRP2 knockdown produced similar effects (Figure 2B). To confirm a functional effect of TfR1 downregulation in sh-IRP2 cells, we measured uptake of fluorophore-labeled transferrin by flow cytometry in LNCaP shControl and sh-IRP2 cells. As expected, LNCaP sh-IRP2 cells exhibited reduced transferrin uptake compared to shControl cells (Figure 2C), demonstrating a diminished ability to import transferrin-bound iron. To further confirm that IRP2 knockdown reduced iron retention in prostate cancer cells, we measured the labile iron pool (LIP) following IRP2 knockdown in LNCaP and PC3 cells. Consistent with our other findings, IRP2 knockdown significantly depleted LIP levels in both cell lines (Figure 2D and 2E), confirming that overexpression of IRP2 drives iron retention in prostate cancer cells.

### Reduction in IRP2 inhibits prostate cancer cell proliferation *in vitro*

**Figure 3:**
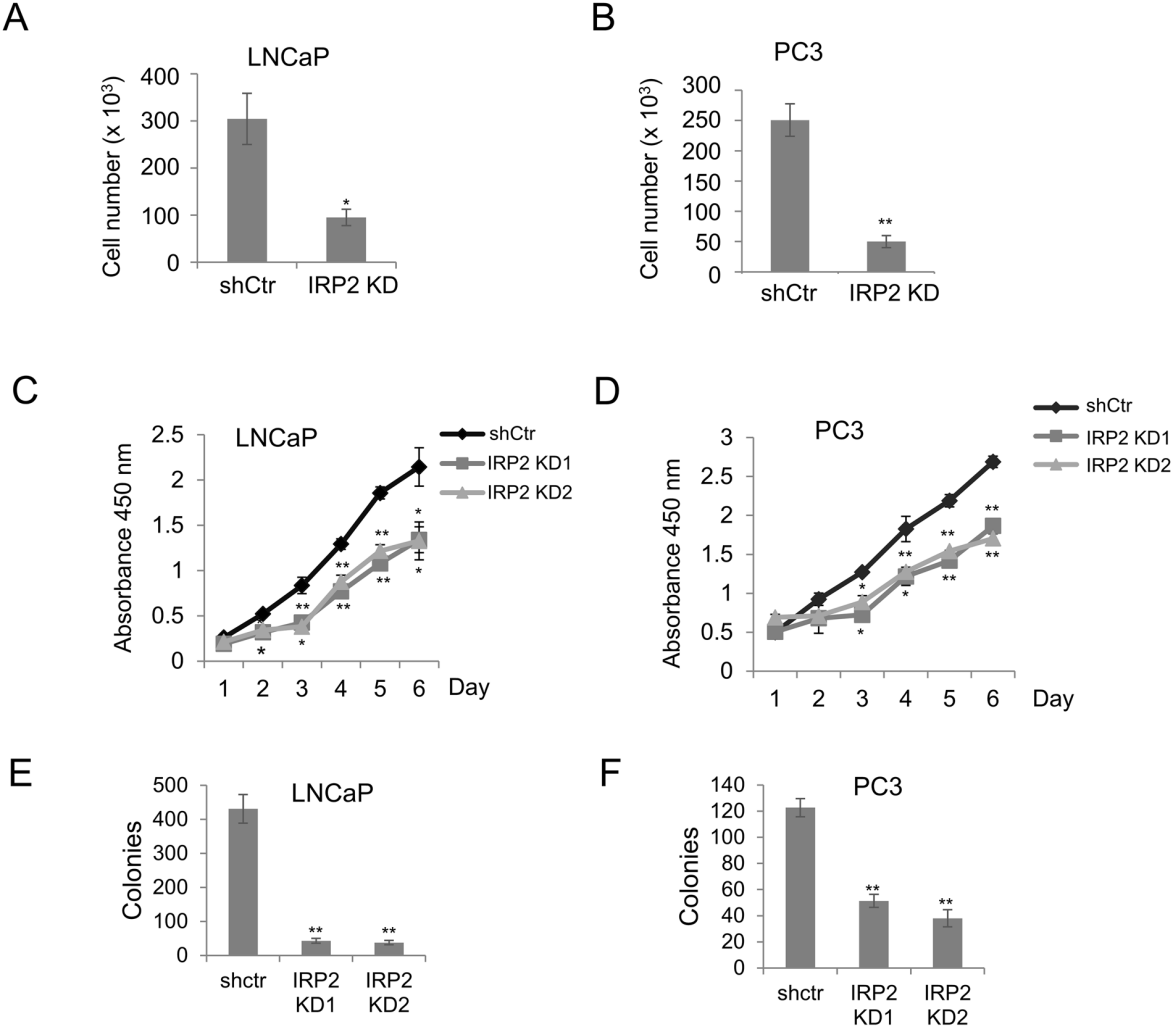
IRP2 knockdown inhibits proliferation of prostate cancer cells **(A, B)** Cell count by hemocytometer of (A) LNCaP and (B) PC3 shCtr and IRP2 KD cells after 7 days. **(C, D)** WST-1 proliferation assay of (C) LNCaP and (D) PC3 shCtr and IRP2 KD cells. **(E, F)** Clonogenic assays for (E) LNCaP (10 days) and (F) PC3 cells (12 days). Data are representative of 3 independent experiments (* p < .05, ** p < .01).

We next sought to determine the role of IRP2 overexpression in prostate cancer cell proliferation. We compared proliferation rates in control and IRP2-depleted cells by direct cell counts. As shown in Figure 3A and 3B, reduction of IRP2 suppressed proliferation in LNCaP and PC3 prostate cancer cells. These results were confirmed using metabolic (WST-1) (Figure 3C and 3D) and clonogenic assays (Figure 3E and 3F). Thus, IRP2 overexpression facilitates prostate cancer cell proliferation.

### IRP1 knockdown has minimal effects on prostate cancer cell proliferation

**Figure 4:**
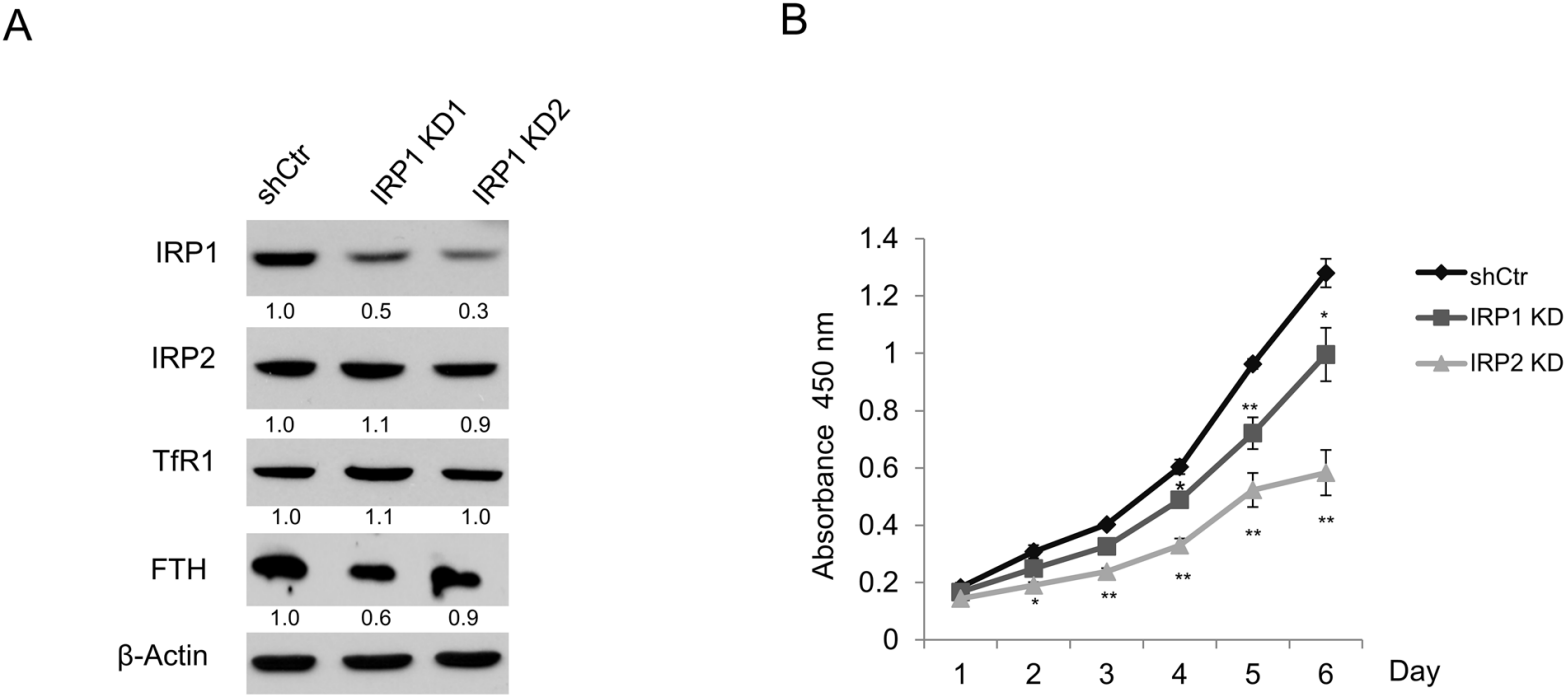
IRP1 silencing does not affect expression of iron proteins and only modestly inhibits proliferation of LNCaP cells **(A)** Western blot of iron regulatory protein 1 (IRP1), iron regulatory protein 2 (IRP2), transferrin receptor 1 (TfR1), ferritin H (FTH), and β-actin (loading control) in LNCaP cells infected with lentiviral IRP1-shRNAs (IRP1 KD1 and KD2) and scrambled control shRNA (shCtr). A polyclonal FTH antibody with increased sensitivity was used for this experiment [47]. **(B)** WST-1 proliferation assay of LNCaP shCtr, IRP1 KD, and IRP2 KD cells. Data are representative of 3 independent experiments (* p < .05, ** p < .01).

These data, supported by previous *in vivo* and cellular studies [15, 16], suggested that IRP2, rather than IRP1 plays a predominant role in regulation of iron metabolism. To directly evaluate the contribution of IRP1 in regulating prostate cancer iron metabolism and cell growth, we utilized two distinct shRNAs to knockdown IRP1 in LNCaP cells. As shown in Figure 4A, there were no appreciable changes in TfR1, FTH, or IRP2 protein following IRP1 knockdown. We then assessed the effects of IRP1 knockdown on cell growth as compared to shControl and sh-IRP2 cells (Figure 4B). IRP1 knockdown led to a modest reduction in cell proliferation rate compared to IRP2 knockdown. These results support a greater dependence of prostate cancer cell growth on IRP2 than IRP1. Further, it is unlikely that the effect of IRP1 knockdown on cell growth was a result of altered iron metabolism, since manipulation of IRP1 did not alter expression of other iron proteins (Figure 4A).

### IRP2 knockdown regulates cell cycle in prostate cancer cells

**Figure 5:**
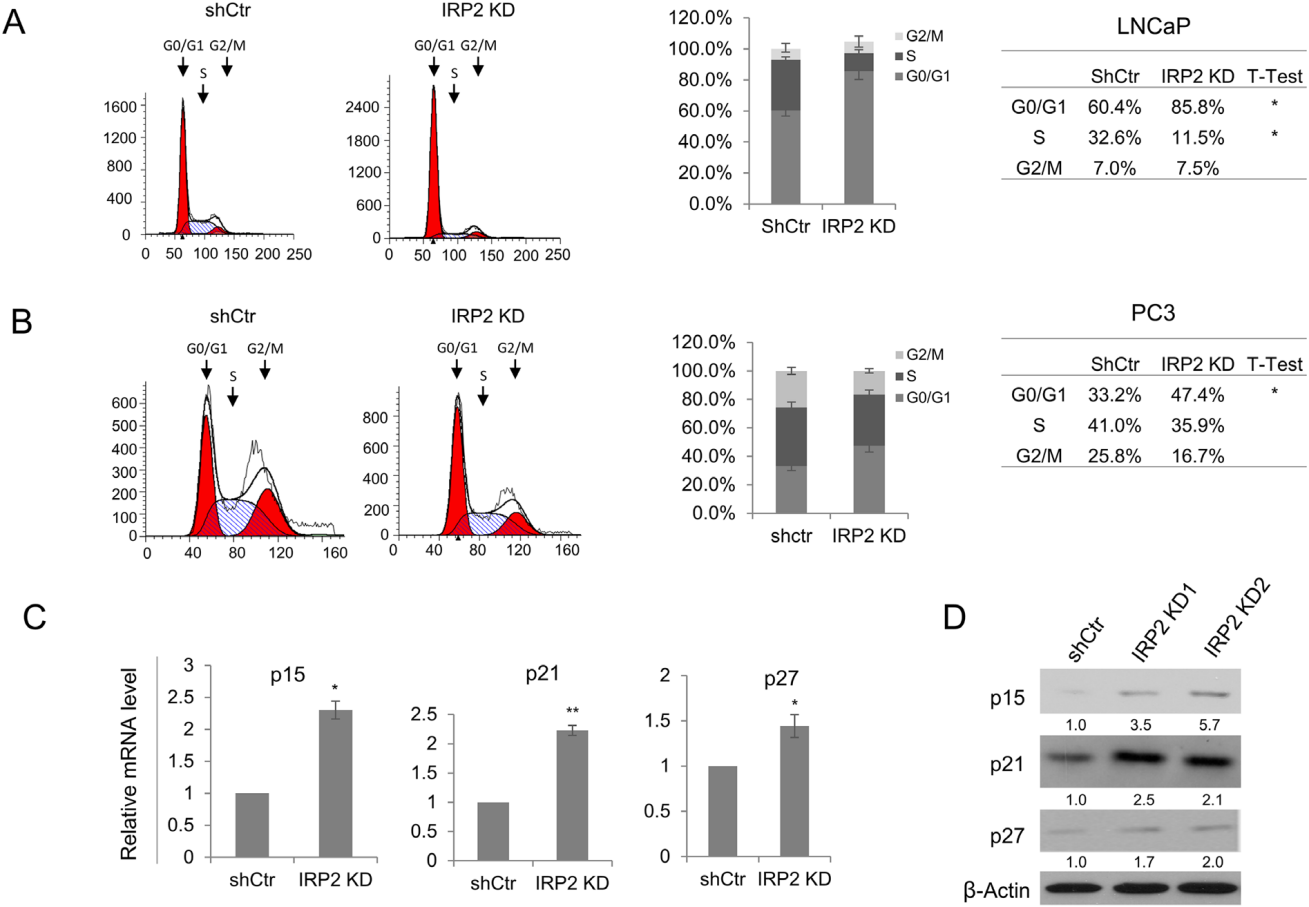
IRP2 knockdown modulates cell cycle regulators and inhibits cell cycle progression **(A, B)** DNA content of propidium iodide stained (A) LNCaP and (B) PC3 control (shCtr) and IRP2 knockdown (IRP2 KD) cells assessed by flow cytometry. Cell cycle distribution was analyzed by ModFit LT software. **(C)** Relative mRNA levels of p15, p21, and p27 in LNCaP shCtr and IRP2 KD cells assessed by real-time qPCR. **(D)** Western blot of p15, p21 and p27 in LNCaP shCtr and IRP2 KD cells. Data are representative of 3 independent experiments (* p < .05, ** p < .01).

Having confirmed that IRP2 knockdown has a pronounced effect on prostate cancer cell proliferation, we sought to identify the mechanism by which cell proliferation is inhibited. We first tested whether iron depletion following IRP2 knockdown resulted in cell cycle inhibition. We labeled control and IRP2 knockdown LNCaP and PC3 cells with propidium iodide and examined cell cycle phase distribution using flow cytometry (Figure 5A and 5B). In both cell lines we observed a significant accumulation of cells in G0/G1 phase following IRP2 knockdown. In LNCaP IRP2 knockdown cells, the increase of cells in G0/G1 phase was accompanied by a significant decrease in cells in S phase (Figure 5A). Similarly, a decrease in the number of cells in S phase was observed following IRP2 knockdown in PC3 cells, although the decrease was not statistically significant. PC3 cells also demonstrated a small decrease in the number of cells in G2/M following IRP2 knockdown (Figure 5B).

Despite some differences, IRP2 knockdown in both LNCaP and PC3 cells resulted in accumulation of cells in G0/G1. To determine the mechanism responsible for G0/G1 arrest in IRP2 knockdown cells, we examined transcript levels of the G0/G1 cell cycle checkpoint proteins p15 (CDKN2B), p21 (CDKN1A), and p27 (CDKN1B) by real-time qPCR. As shown in Figure 5C, these cell cycle regulating genes were upregulated following IRP2 knockdown in LNCaP cells, consistent with cell cycle inhibition. Upregulation of p15, p21 and p27 protein following IRP2 knockdown in these cells was confirmed by western blot (Figure 5D).

While many genes regulated by the IRP-IRE system are involved in iron metabolism, IRPs have also been reported to regulate genes involved in other cellular processes [17]. To confirm that the effects of IRP2 knockdown on cell growth and p15, p21, and p27 were caused by iron depletion, we tested whether these changes could be mimicked by depleting iron with the iron chelator desferoxamine (DFO). LNCaP cells treated with DFO showed a dose-dependent reduction in cell proliferation (Supplementary Figure 1A) and recapitulated the induction of p15, p21, and p27 (Supplementary Figure 1B). Taken together, these data indicate that prostate cancer cells overexpress IRP2 to maintain adequate iron levels to support rapid cell cycling. When IRP2 is reduced, prostate cancer cell proliferation is impaired by cell cycle inhibitors p15, p21, and p27.

### Apoptosis is induced by IRP2 depletion in prostate cancer cells

**Figure 6:**
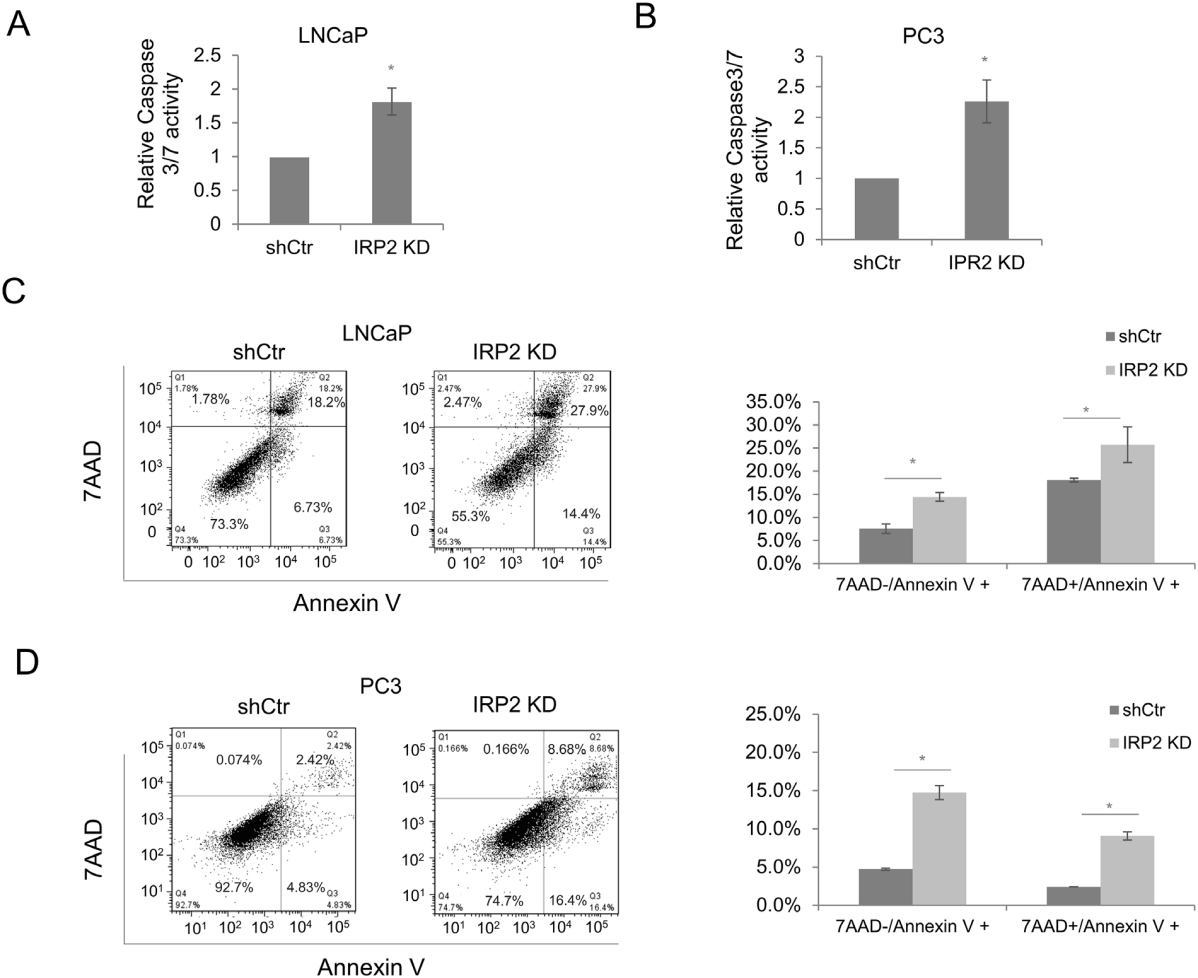
IRP2 knockdown induces apoptosis **(A, B)** Caspase-Glo 3/7 assay in (A) LNCaP and (B) PC3 control (shCtr) and IRP2 knockdown (IRP2 KD) cells. **(C, D)** Annexin V and 7-aminoactinomycin D (7AAD) staining in (C) LNCaP and (D) PC3 cells shCtr and IRP2 KD cells measured by flow cytometry. Bar graphs indicate the quantification of early apoptotic (7AAD-/Annexin V+) and late apoptotic cells (7AAD+/Annexin V+) (* p < .05).

Cell cycle checkpoint proteins are involved in both cell cycle regulation and initiation of apoptotic death. As iron deficiency has been shown to induce apoptosis [18], we sought to determine if IRP2 depletion also induced apoptosis of prostate cancer cells. Visual inspection of cell cultures revealed an increase in detached cells, a sign of cell death, following IRP2 knockdown in prostate cancer cells. To test if IRP2 knockdown triggered apoptosis, we measured the activity of caspase 3/7 in LNCaP and PC3 cells. We found that IRP2 knockdown increased caspase 3/7 activity in both cell lines (Figure 6A and 6B). We next determined the proportion of apoptotic cells by flow cytometry using 7-AAD/Annexin V staining [19]. As demonstrated in Figure 6C and 6D, Annexin V single-stained (early apoptotic) and Annexin V/7AAD double-stained (late apoptotic) cells were both significantly increased following IRP2 knockdown in LNCaP and PC3 cells. These results indicate that IRP2 overexpression is an important determinant of cell fate that allows prostate cancer cells to avoid apoptosis.

### IRP2 depletion attenuates the growth of prostate cancer xenografts

**Figure 7:**
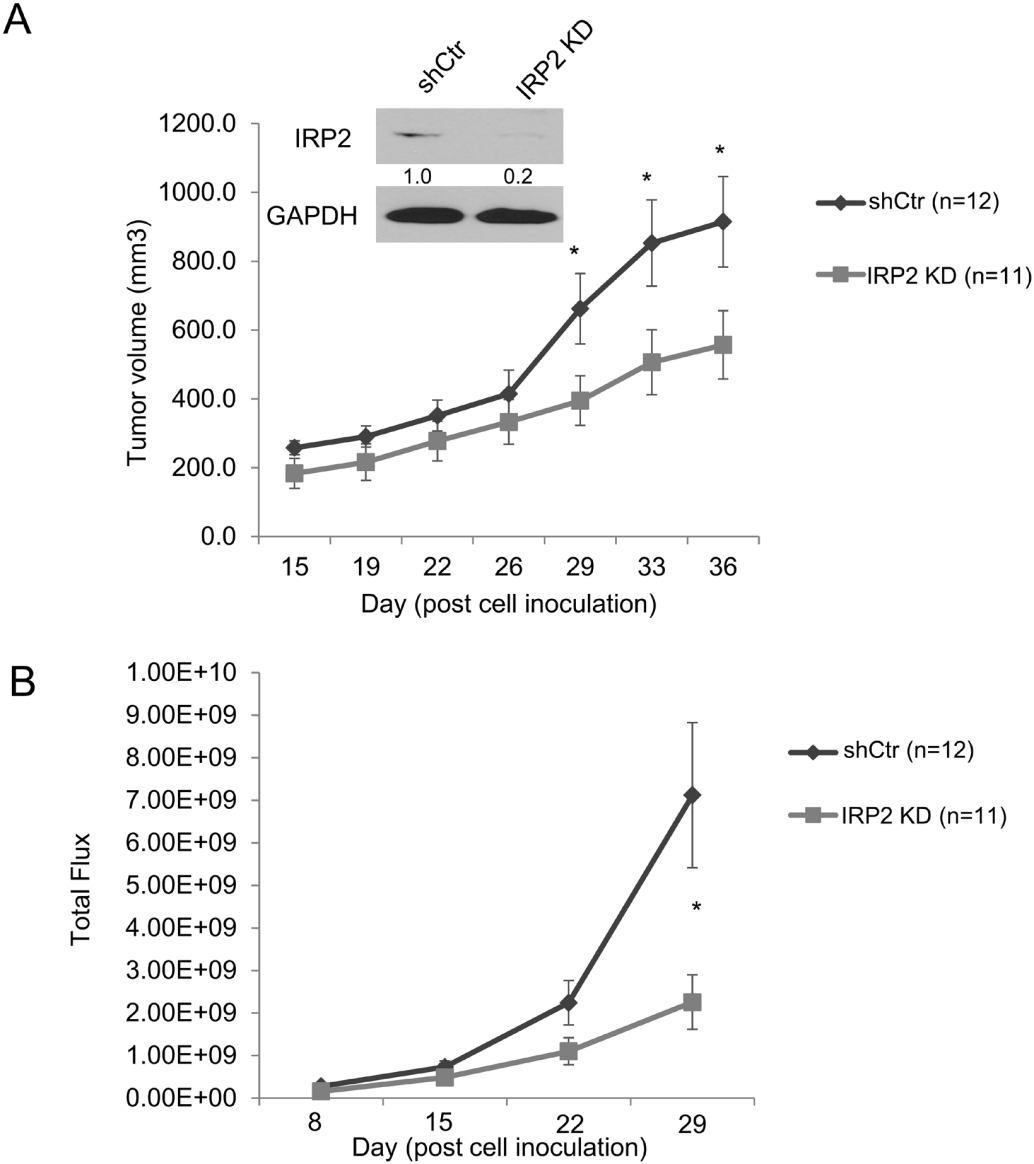
IRP2 deficiency inhibits tumor growth of prostate cancer cells *in vivo* Luciferase expressing PC3 (PC3/Luc) cells were infected with lentiviral scrambled control shRNA (shCtr) or IRP2-shRNA (IRP2 KD). 2 × 10^6^ PC3/Luc cells were inoculated into flanks of nude mice by subcutaneous injection. **(A)** Western blot of iron regulatory protein 2 (IRP2) and GAPDH (loading control) at day 0. Tumor growth was evaluated by caliper measurement at 15, 19, 22, 26, 29, 33, and 36 days post-inoculation. **(B)** Tumor growth was evaluated by bioluminescent luciferase imaging at 8, 15, 22, and 29 days post-inoculation. Data are means ± Standard Error with n=11/12 (* p < .05).

We next utilized a xenograft model to assess the role of IRP2 in prostate cancer growth *in vivo*. PC3 cells expressing a stable luciferase construct (PC3/Luc) were generated to facilitate tracking of tumor cells *in vivo*. These cells were then infected with sh-IRP2 or shControl lentiviruses. We confirmed knockdown of IRP2 in PC3/Luc sh-IRP2 cells prior to starting the experiment (Figure 7A). PC3/Luc shControl and sh-IRP2 cells were inoculated into the flanks of nude mice and tumors allowed to grow for 15 days prior to initiating measurements of tumor size. IRP2 knockdown significantly reduced tumor volume beginning at day 29 as assessed by both caliper measurement (Figure 7A) and bioluminescent imaging (Figure 7B). These findings show that IRP2 depletion can suppress prostate tumor growth *in vivo*.

## DISCUSSION

Growing evidence demonstrates that iron metabolism is remodeled in numerous cancer types to enable enhanced iron acquisition and retention [9, 12, 13]. Indeed, a recent bioinformatics analysis revealed that expression of nearly half of 61 genes involved in some aspect of iron metabolism were associated with distant metastasis-free survival in breast cancer [20]. While these findings suggest that iron dysregulation promotes tumor progression, the mechanisms by which alterations in iron metabolism are implemented and their effects on tumor behavior remain incompletely understood.

We therefore explored the role of the only two known mammalian IRPs, IRP1 and IRP2, which are master regulators of cellular iron. IRP1 and IRP2 share an overall amino acid identity of approximately 61% [5] and both have high affinity for IREs. Despite these similarities, IRP1 and IRP2 have been reported to execute different cellular functions through regulation of non-overlapping mRNA targets [17, 21]. Our findings support a functional difference between IRP1 and IRP2 by demonstrating that IRP2 is specifically co-opted by prostate cancer cells to alter normal iron homeostasis and retain increased intracellular iron (Figure 2). IRP1 was unable to alter ferritin and TfR1 when depleted by shRNA knockdown (Figure 4), suggesting that the modest effect of IRP1 knockdown on cell growth is likely iron-independent. In contrast, IRP2 knockdown inhibited prostate cancer cell growth in an iron-dependent fashion (Figures 2 and 3). These findings are consistent with previous reports that IRP2 plays a predominant role over IRP1 in iron homeostasis *in vitro* [16] and *in vivo* [15].

Our findings demonstrate that constitutive overexpression of IRP2 is broadly observed in prostate cancer. Overexpression of IRP2 was consistently observed in all prostate cancer cell lines studied (Figure 1). IRP2 protein is tightly regulated by FBXL5-mediated proteasomal degradation [22–24] as well as non-proteasomal degradation [25]. While it is possible that prostate cancer cells drive IRP2 overexpression by altering post-transcriptional regulation, our findings demonstrate that IRP2 is transcriptionally upregulated in prostate cancer cells (Figure 1). We speculate that *c-myc*, an oncogene that is frequently misregulated in prostate cancer [26, 27] and is a known transcriptional regulator of IRP2 [28], may be the driver of IRP2 overexpression in some prostate tumors. Future experiments will be required to assess whether this or other mechanisms drive IRP2 overexpression in prostate cancer.

Overexpression of IRP2 may enable prostate cancer cells to bypass normal feedback loops that are designed to limit iron uptake in cells that are iron replete. In non-cancer cells, IRPs are inactivated under conditions of high iron, triggering a compensatory decrease in TfR1 and increase in ferritin [14]. Prostate cancer cells that overexpress IRP2 bypass this checkpoint and express high levels of TfR1 and low levels of ferritin despite high levels of intracellular iron (Figure 1). Overexpression of IRP2 is thus a mechanism enabling the increased iron uptake and retention that characterizes cancer cells. To test the dependence of prostate cancer cells on this pathway, we depleted IRP2 in prostate cancer cells using lentiviral shRNA. IRP2 knockdown dramatically altered the iron regulatory proteins TfR and ferritin, reducing intracellular iron (LIP) levels (Figure 2). IRP2-induced LIP reduction was sufficient to inhibit prostate cancer cell proliferation, both *in vitro* (Figure 3) and *in vivo* (Figure 7), and induce apoptosis (Figure 6).

Because cell cycle checkpoints link cell cycle progression and apoptotic cell death [29], we investigated the effects of IRP2 knockdown on these proteins. We determined that IRP2 knockdown induced cell cycle checkpoint proteins p15, p21, and p27 (Figure 5C and 5D), consistent with the observed G0/G1 cell cycle arrest (Figure 5A and 5B).

While IRP2 knockdown resulted in G0/G1 arrest in both LNCaP and PC3 cells, the magnitude of cell cycle arrest differed, as did the distribution of cells in S and G2/M phases. Thus, LNCaP IRP2 KD cells exhibited a robust G0/G1 arrest and an attendant decrease in the percentage of cells in S phase, whereas PC3 IRP2 KD cells showed a moderate G0/G1 arrest and a decrease in the percentage of cells in G2/M phase (Figure 5). It is likely that the observed differences in the effects of IRP2 on the cell cycle in these two cell lines is a result of differences in their genetic backgrounds, since LNCaP and PC3 cells derive from quite different prostate tumors [30–32]. In particular, LNCaP cells express wildtype-p53, whereas PC3 cells lack functional p53 due to a nonsense mutation [33]. p53 is known to induce G0/G1 arrest and can also play a role in G2/M arrest [34]. p53 was induced by IRP2 knockdown in LNCaP cells (Supplementary Figure 2), suggesting that p53 status may contribute to the enhanced G0/G1 arrest observed in LNCaP cells when compared to PC3 cells. Other differences between these two cell lines may also play a role [35]. Importantly, these findings demonstrate that iron depletion by IRP2 overexpression is an effective method for inhibiting growth of biologically diverse prostate cancer cell types.

Given the demand of cancer cells for iron, iron depletion may be a promising therapeutic strategy to restrict tumor growth. Iron chelators are currently being evaluated as anti-cancer agents [36], as are iron-targeting gallium compounds [37]. Our finding that IRP2 knockdown in prostate cancer cells recapitulates the effects of iron chelators on cell cycle inhibition [38, 39] (Figure 5) and induction of apoptosis [40–43] (Figure 6) suggests that targeting IRP2 may represent an alternative approach to restricting tumor growth by targeting iron. The substantial IRP2 overexpression in cancer cells suggests that there may be a favorable therapeutic window for IRP2-targeted treatments. Further, because IRP2-targeted therapy has the potential to alter multiple facets of cellular iron homeostasis, including iron uptake, storage, utilization, and export, inhibiting IRP2 may be more effective than blocking individual components of iron metabolism, such as TfR1 [44]. For these reasons, IRP2 may represent a promising target for cancer therapy.

## MATERIALS AND METHODS

### Cell culture

Cell culture reagents were obtained from Gibco (Life Technologies, Carlsbad, CA) unless otherwise specified. The human prostate cancer cell lines LNCaP, PC3, and DU145 were purchased from American Type Culture Collection (ATCC). Cells were grown in RPMI 1640 medium containing 10% fetal calf serum and 1% PenStrep and incubated at 37°C in a humidified atmosphere containing 5% CO_2_. VCaP cells were provided by Dr. Donna Peehl (Stanford University) and cultured in Dulbecco's Modified Eagle's Medium (DMEM) containing 10% fetal calf serum and 1% PenStrep. Normal prostate epithelial cells (PrEC) were purchased from Lonza and cultured in Prostate Cell Growth Medium (Lonza, CC-3166). Cells were studied at a low passage number (less than 30 thaw-freeze cycles after thaw from early-passage frozen stocks). Cell authentication was performed by ATCC based on short-tandem-repeat (STR) profiling and cell morphological features were routinely monitored.

### DNA vectors

Lentiviral shRNA constructs were constructed on a previously described shRNA vector backbone using an established protocol [45, 46]. Targeted shRNA vectors were generated using the following target sequences: 5’-GATCTTACAGTTGACCATTCT-3’ (shRNA-IRP2-#1), 5’-GGAGTGGCTGGAAAGTTTGTT -3’ (shRNA-IRP2-#2), 5’-GTAATAGCATATGCAATTGCT-3’ (shRNA-IRP1-#1), and 5’-GAACGATACACTATCATTATT-3’ (IRP1-shRNA-#2). The non-targeted, scrambled shRNA control was previously validated [46]. A lentiviral luciferase expression vector was constructed by subcloning the LUC2 gene and zeocin resistance gene from the pGL4 vector (Promega Co., Madison, WI, USA) into the pLKO.1-shCtr vector (Addgene catalog #10879) (34). First, the shRNA expression cassette was removed and the puromycin resistance gene was supplanted with the LUC2 coding region, an internal ribosome entry site, and the zeomycin resistance gene. These modifications resulted in a vector expressing luciferase and zeocin driven by the original phosphoglycerate kinase (PGK) promoter. shRNA knockdown experiments were performed 5-7 days after infection.

### Cell proliferation assays

Cell proliferation was measured manually by hemocytometer cell counting with trypan blue staining to exclude non-viable cells. 8-16 quadrants were counted. Individuals performing these measurements were not blinded to the identity of the samples. A colorimetric assay using the tetrazolium salt 4-[3-(4-lodophenyl)-2-(4-nitrophenyl)-2H-5-tetrazolio]-1,3-benzene disulfonate (WST-1) (Roche) was also used to measure cellular proliferation following the manufacturer's instructions. Clonogenic assays were performed for three days following lentiviral shRNA infection. Cells were seeded in 6-well plates at densities of 800 (LNCaP) or 400 (PC3) cells per well. Cells were then fixed in 10% formalin after 10 days (LNCaP) or 12 days (PC3) and stained with 0.25% crystal violet to detect colony formation.

### Cell cycle analysis

Cells were harvested and washed twice with PBS and the cell pellets were suspended at a concentration of 1 × 10^6^ cells/ml. Cells were then gently vortexed to minimize clumping, meanwhile three volumes of cold (-20°C) absolute ethanol were added to fix the cells at -20°C for at least 1 hour. Fixed cells were further washed twice with PBS and mixed with an appropriate volume of FxCycle™ PI/RNase Staining Solution (Life Technologies, Carlsbad, CA) to stain nuclear DNA. Stained cells were maintained at room temperature for 15-30 minutes to ensure complete staining and minimize RNA contamination. Cell cycle analysis was performed on a MACSQuant flow cytometer (Miltenyi Biotec, Bergisch Gladbach, Germany). The data were analyzed with ModFit LT 3.0 software (Verity Software House, Topsham, ME, USA).

### Apoptosis assay

The Caspase-Glo 3/7 assay kit (Promega) was used to assess caspase activity. Cell apoptosis was quantified using a MACSQuant flow cytometer following double staining with the APC-Annexin V and 7-aminoactinomycin D (7-AAD) detection kit (BD Biosciences).

### Western blotting and quantitative reverse transcription PCR (RT-qPCR)

To detect cellular protein levels, cell pellets were harvested and lysed by sonication in RIPA buffer (25mM Tris-HCl pH 7.6, 150mM NaCl, 1% NP-40, 1% sodium deoxycholate, 0.1% SDS, plus 1x protease inhibitor cocktail). Lysate protein concentration was measured using a Pierce BCA Protein Assay kit (Life technologies). Approximately 40-60 μg of protein for each sample was resolved on a 10% or 12% SDS-PAGE gel and then transferred onto a nitrocellulose membrane for western blotting. The following primary antibodies were used for detection: glyceraldehyde-3-phosphate dehydrogenase (GAPDH) (Fitzgerald, 10R-G109a), TfR1 (Life Technology, 13-6800), ferritin H monoclonal (hybridoma; Harlan Bioproducts for Science) and polyclonal [47], IRP1 (MediMabs, MM-0074), IRP2 (Santa Cruz Biotechnology, sc-33682), Beta Actin (Abcam, ab6276), p15 (Santa Cruz Biotechnology, SC612), p21 (Becton Dickinson, 556430), p27 (Cell Signaling Technology, #3698)), p53 (Calbiochem Research Biochemicals, OP43). Goat anti-rabbit-HRP (Bio-Rad Laboratories, 170-6515) and goat anti-mouse-HRP (Bio-Rad Laboratories, 170-6516) secondary antibodies were used.

RT-qPCR experiments were conducted essentially as described previously [13]. Briefly, RNA was extracted using the High Pure RNA Isolation Kit (Roche). cDNA was produced using the TaqMan® Reverse Transcription kit (Applied Biosystems). RT-qPCR was performed on a ViiA™ 7 Real-Time PCR System (Applied Biosystems) using a SYBR green-based PCR method. Primer sequences are listed in Supplementary Table 1. Experiments were performed 3 times with samples in triplicates. Representative results are shown. Comparative Ct method was used to calculate relative gene expression.

### Labile iron pool (LIP) assay

The labile iron pool was measured using calcein AM (2 μM, 15 min incubation) as a fluorescent probe for iron content. The concentration of calcein-bound iron was determined following addition of salicyladehyde-isonicotinoyl-hydrazone (10 μM) essentially as described [48].

### Transferrin binding assay

To perform the transferrin binding assay, LNCaP cells were incubated with 50 μg/ml Alexa Fluor 647 labeled transferrin (Life Technologies). After a 20 minute incubation, cells were gently trypsinized and washed 3 times with 1x PBS to minimize non-specific binding of fluorescent transferrin. The fluorescent intensity of the harvested cells was determined by flow cytometer.

### Xenograft experiments

Animal protocols were approved by the Institutional Animal Care and Use Committee (IACUC) at the University of Connecticut Health Center. 2 × 10^6^ PC3-Luc cells (infected with scrambled control or IRP2 shRNA lentiviruses) were suspended in 200 μl of matrigel and injected subcutaneously into the rear flank of athymic nude mice (The Jackson Lab). Twelve mice were used in each group, however one mouse was excluded from the IRP2 knockdown group due to a failure to form a tumor. Tumor growth was monitored by digital calipers and bioluminescent luciferase imaging using an IVIS Spectrum (Perkin Elmer). Bioluminescent luciferase imaging was acquired and analyzed using Living Imaging 4.4 software (Perkin Elmer). Tumor volumes (V) were calculated by the formula V = π/6 ×L × W^2^ (L: length, W: width).

### Statistical analyses

*In vitro* experiments were performed at least three times. Experimental data are presented as mean ± SD, unless otherwise indicated. Statistical analysis was performed using GraphPad Prism (Graphpad Software). Student's t test (two-tailed) was used to evaluate the significance of observed differences between two groups. A p-value <0.05 was considered statistically significant (* p < .05, ** p < .01, *** p < .001).

## SUPPLEMENTARY MATERIALS FIGURES AND TABLE



## Abbreviations

7-AAD: 7-aminoactinomycin D
DFO: desferoxamine, an iron chelator
FPN: ferroportin
FTH: ferritin heavy chain
GAPDH: glyceraldehyde-3-phosphate dehydrogenase
IACUC: Institutional Animal Care and Use Committee
IREs: iron responsive elements
IRPs: iron-responsive element-binding proteins
KD: knockdown
Luc: luciferase
p15: cyclin dependent kinase inhibitor 2B
p21: cyclin-dependent kinase inhibitor 1A
p27: cyclin dependent kinase inhibitor 1B
p53: tumor protein p53
PrEC: normal prostate epithelial cells
RT-qPCR: quantitative reverse transcription polymerase chain reaction
shRNA: short hairpin RNA
shCtr: shRNA control
TfR: transferrin receptor
UTR: untranslated region
WST-1: water soluble tetrazolium salt-1
