# Supplementary material for: Iron-responsive element-binding protein 2 plays an essential role in regulating prostate cancer cell growth
Source: Oncotarget. 2017 Jul 17;8(47):82231–43. doi: 10.18632/oncotarget.19288 (PMC5669885; doi:10.18632/oncotarget.19288)
Supplement: Supplementary file 1 [file oncotarget-08-82231-s001.pdf]

Iron-responsive element-binding protein 2 plays an essential role in regulating prostate cancer cell growth

SUPPLEMENTARY MATERIALS

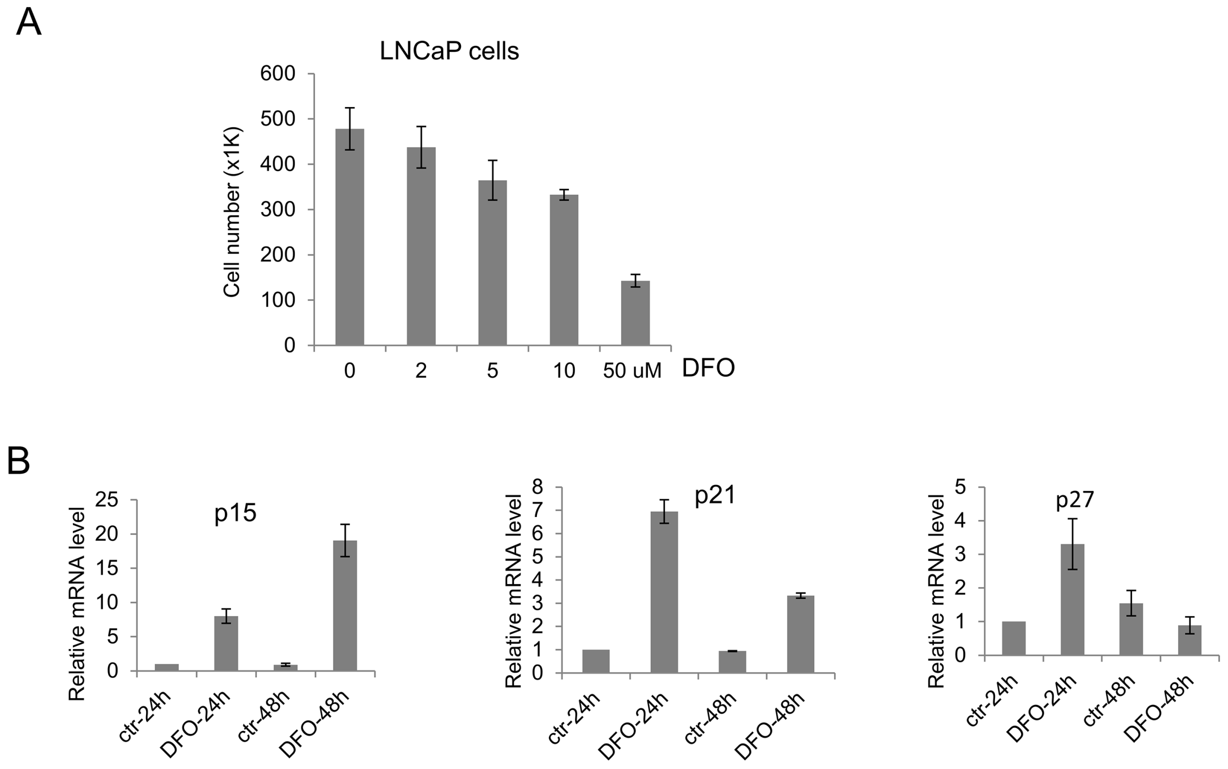

Supplementary Figure 1: Iron chelator (DFO) reduces prostate cancer cell growth and induces expression of cell cycle inhibitors. (A) Desferoxamine (DFO) inhibits LNCaP cell growth in a dose dependent manner. LNCaP cells were treated with various doses of DFO and cells were counted 6 days after treatment. **(B)** Real-time qPCR detection of cell cycle regulators, p15, p21 and p27, in LNCaP cells ± DFO.

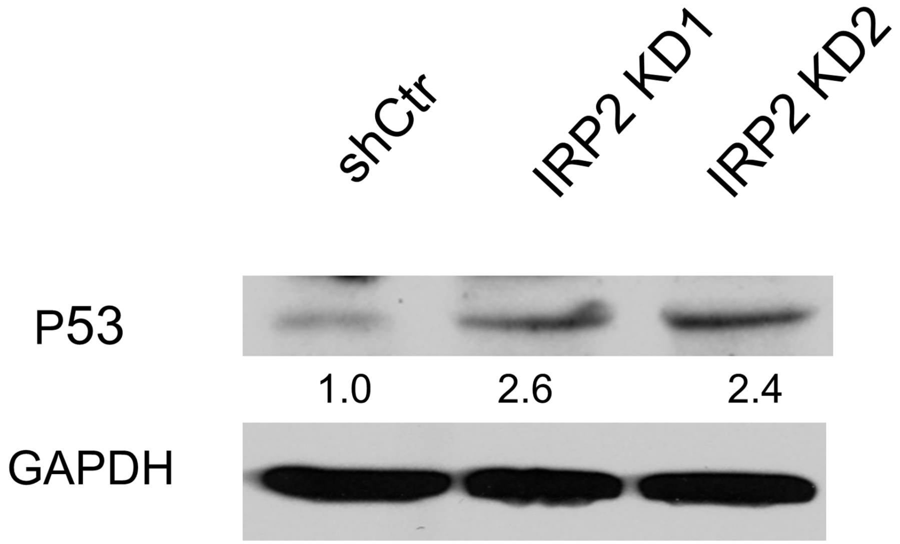

Supplementary Figure 2: p53 level in IRP2 knockdown LNCaP cells. Western blots were performed to detect p53 expression.

Supplementary Table 1: Real-time PCR primer sets

Genes	Primer sequences (upper)	Primer sequences (lower)
IRP2	accagaggtggttggatgtgagtt	actcctacttgcctgaggtgcttt
IRP1	tgcttcctcaggtgattggctaca	tagctcggtcagcaatggacaact
P21	cgctctacatcttctgccttagtc	gaacctctcattcaaccgcctag
P27	aaccgacgattcttctactc	atgtatatcttccttgcttcatc
P15	atcccaacggagtcaacc	accagcgtgtccaggaag
GAPDH	gaaggtgaaggtcggagtc	gaagatggtgatgggatttc
β-Actin	ttgccgacaggatgcagaagga	aggtggacagcgaggccaggat
